# Infrequent Denture Cleaning Increased the Risk of Pneumonia among Community-dwelling Older Adults: A Population-based Cross-sectional Study

**DOI:** 10.1038/s41598-019-50129-9

**Published:** 2019-09-24

**Authors:** Taro Kusama, Jun Aida, Tatsuo Yamamoto, Katsunori Kondo, Ken Osaka

**Affiliations:** 1 Department of International and Community Oral Health, Tohoku University Graduate School of Dentistry, Aoba-ku, Sendai, Miyagi Japan; 2 Department of Disaster Medicine and Dental Sociology, Graduate School of Dentistry, Kanagawa Dental University, Yokosuka, Kanagawa Japan; 3 Department of Social Preventive Medical Sciences, Center for Preventive Medical Sciences, Chiba University, Chuo Ward, Chiba-shi, Chiba Japan; 4 Department of Gerontological Evaluation, Center for Gerontology and Social Science, National Center for Geriatics and Gerontology, Obu city, Aichi Japan

**Keywords:** Gerodontics, Removable prosthodontics

## Abstract

Pneumonia is a leading cause of death among older adults. The effectiveness of oral care in preventing pneumonia in nursing homes and hospitals has been reported. However, in community-dwelling older adults, the role of denture cleaning in preventing pneumonia remains unknown. We aimed to investigate the association between infrequent denture cleaning and the risk of pneumonia in community-dwelling older adults. This cross-sectional study was based on the self-reported questionnaire targeting towards community-dwelling older adults aged ≥65 years. Responses of 71,227 removable full/partial denture users were included. The incidence of pneumonia within the last one-year and the frequency of denture cleaning (daily/non-daily) were treated as dependent and independent variables, respectively. The odds ratio (OR) and 95% confidence interval (CI) were calculated by the inverse probability weighting (IPW) method based on the logistic regression model. The mean age of the participants was 75.2 ± 6.5 years; 48.3% were male. Overall, 4.6% of the participants did not clean their dentures daily; 2.3% and 3.0% who did and did not clean their dentures daily, respectively, experienced pneumonia. After IPW, infrequent denture cleaning was significantly associated with pneumonia incidence (OR = 1.30, 95% CI = 1.01–1.68)). This study suggests that denture cleaning could prevent pneumonia among community-dwelling older adults.

## Introduction

Pneumonia is a leading cause of hospitalization and death^1,2^. It is widely prevalent among the older population because of the decline in immune system and respiratory function with advancing age^3^. Aspiration is one of the mechanisms that explains the onset of pneumonia among older adults^4,5^. In fact, oral bacteria have been identified in the lungs of the patients who developed pneumonia; therefore, a relationship between aspiration of oral bacteria and pneumonia is strongly suggested^6^. In addition, because a substantial proportion of older adults are affected by dysphagia^7,8^, the risk of pneumonia through aspiration may increase^4^. To reduce the risk of aspiration pneumonia, oral care has been implemented in nursing homes and has successfully decreased the incidence of pneumonia among the nursing home residents^9–11^.

Although the role of oral care in reducing the risk of pneumonia has been recognized, the importance of denture cleaning has been relatively neglected. The presence of fewer or no teeth is prevalent among the older adults^12^; therefore, the use of removable dentures is a common treatment option. On the surface of the denture, a biofilm composed of microorganisms called “denture plaque” rapidly develops upon insertion after cleansing^13^. There is a possibility that the denture plaque may reach the lungs by aspiration, causing aspiration pneumonia. Although generally included as a part of oral care^11^, previous studies have not focused on denture cleaning alone. In addition, most of the previous studies on the relationship between oral hygiene and pneumonia were carried out in nursing homes and hospitals^14,15^. However, the risk of aspiration pneumonia is considered to be high in community-dwelling older adults. To the best of our knowledge, no study has investigated the association between denture cleaning and pneumonia among community-dwelling older adults. From a public health viewpoint, as the majority of older adults are community-dwellers and not institutionalized, the prevention of pneumonia among community-dwelling older adults is important. In this study, we investigate whether infrequent denture cleaning is associated with the risk of developing pneumonia among community-dwelling older adults.

## Methods

### Settings and participants

This cross-sectional study is based on a self-reported questionnaire. The data were obtained from the survey of the 2016 Japan Gerontological Evaluation Study (JAGES). JAGES targeted the community-dwelling older adults aged ≥65 years, who were not certified to be eligible for long term public care. Information on social, behavioral, and health factors were collected. JAGES in 2016 was conducted in 39 municipalities in Japan. The questionnaire was sent by post and was retrieved by mail.

### Dependent variable

We used the self-reported incidence of pneumonia within the last one-year as a dependent variable. We asked the question “*Did you experience the following diseases within the last one year?*” Those who answered “*pneumonia*” were considered to be the individuals who suffered from pneumonia within the last one year.

### Independent variable

We used the frequency of denture cleaning as an independent variable. To those who used removable dentures, we asked the question “*Do you clean your dentures daily?*”; the choices provided were “*Yes*” or “*No*.” We defined people chose “*Yes*” as those who cleaned their denture daily and “*No*” as those who cleaned their dentures infrequently (non-daily).

### Covariates

We selected possible cofounders as covariates based on previous studies and clinical knowledge^1,16,17^; this included age, sex, smoking status, educational status, equivalent income, number of teeth, activities of daily living (ADL), comorbidity related to stroke or dementia, and experience of pneumococcal vaccination within last five-year.

### Statistical analysis

We estimated the propensity score for the independent variable. The stabilized average treatment effect (ATE) on the risk of pneumonia was calculated using the inverse probability weighting (IPW) method. To predict the propensity score for infrequent denture cleaning, we used the logistic regression model; all the covariates were included as possible confounders and the stabilized ATE weight was calculated. The stabilized ATE weight was used to avoid instability of the estimated effect size due to extreme weighting^18^. We compared the standardized difference between the categories of independent variable before and after stabilized ATE weighting^19,20^. The standardized difference was used to check the balance of the covariates between the treated and control groups. If standardized difference of all covariates was <0.1 after weighting, it was regarded as well balanced. We developed the logistic regression model; the odds ratios (ORs) and 95% confidence intervals (95% CIs) were calculated using IPW with stabilized ATE weights (stabilized ATE-IPW). For missing responses, we presumed that the missing pattern of the original data set was missing at random. Multiple imputation by chained equation (MICE) was used to generate 20 imputed datasets. We calculated the stabilized ATE weighted OR for each data set and combined all estimators by Rubin’s rule^21^. In the sensitivity analysis, the participants were stratified into two age groups (<75 or ≥75 years) for IPW. Then, the interaction effect of age and frequency of denture cleaning was confirmed by using the relative excess risk due to interaction (RERI) as additive scales and the ratio of OR as multiplicative scale between them^22^. We used Stata/MP version 15 (Stata Corp., College Station, TX, USA) for statistical analysis.

### Ethical issue

In this study, the process of obtaining informed consent was as follows: the questionnaire was sent by mail along with the explanation of the study; the participants read the written explanation about the aim of study and replied. Hence, we considered that informed consent was provided by those who replied and sent back the questionnaire. The JAGES protocol in 2016 was approved by the ethics committee of National Center for Geriatrics and Gerontology (No. 992) and the ethics committee of Chiba University (No. 2493). We followed the STROBE Statement to report our observational study.

## Results

From a target population of 279,661, 180,021 individuals participated in the survey (response rate = 70.2%). Of these, 88,994 (49.4%) participants who used removable dentures (including both removable full/partial dentures) were included in this analysis. However, 17,767 participants with missing information regarding the dependent variable were excluded. Finally, data of 71,227 participants were included in the analysis. Table 1 shows the characteristics of the participants. The mean age was 75.2 years (SD = 6.5); 48.3% were male. Overall, 2.3% (n = 1,666) and 97.7% (n = 69,561) of the participants, respectively, did and did not experience pneumonia within the last one year.

**Table 1:** Characteristics of the participants (n = 71,227).

Characteristics	All participants (n = 71,227)		Experienced pneumonia within last one-year (n = 1,666)		Not experienced pneumonia within last one-year (n = 69,561)	
	n	%	n	%	n	%
**Frequency of denture cleaning**						
Daily	67,208	94.4	1,547	92.9	65,661	94.4
Non-daily	3,293	4.6	100	6.0	3,193	4.6
Missing	726	1.0	19	1.1	707	1.0
**Age**						
65–69 years	16,770	23.5	248	14.9	16,522	23.8
70–74 years	18,579	26.1	365	21.9	18,214	26.2
75–79 years	17,347	24.4	425	25.5	16,922	24.3
80–84 years	11,858	16.6	369	22.2	11,489	16.5
≥85 years	6,673	9.4	259	15.6	6,414	9.2
**Sex**						
Male	34,393	48.3	984	59.1	33,409	48.0
Female	36,825	51.7	682	40.9	36,143	52.0
Missing	9	0.0	0	0.0	9	0.0
**Education**						
≤9 years	25,133	35.3	706	42.4	24,427	35.1
10–12 years	28,513	40.0	596	35.8	27,917	40.1
≥13 years	16,611	23.3	331	19.9	16,280	23.4
Missing	970	1.4	33	2.0	937	1.4
**Equivalent income (100 JPY ≈ 1 USD**)						
<1,000,000 JPY	7,568	10.6	230	13.8	7,338	10.6
1,000,0000–1,999,999 JPY	21,017	29.5	455	27.3	20,562	29.6
2,000,0000–2,999,999 JPY	13,401	18.8	274	16.5	13,127	18.9
3,000,0000–3,999,999 JPY	8,055	11.3	124	7.4	7,931	11.4
≥4,000,000 JPY	5,701	8.0	117	7.0	5,584	8.0
Missing	15,485	21.8	466	28.0	15,019	21.6
**Smoking status**						
Never	39,027	54.8	702	42.2	38,325	55.1
Quite	22,368	31.4	772	46.3	21,596	31.1
Current	8,726	12.3	145	8.7	8,581	12.3
Missing	1,106	1.5	47	2.8	1,059	1.5
**Dementia**						
Yes	484	0.7	16	1.0	468	0.7
No	68,468	96.1	1,620	97.2	66,848	96.1
Missing	2,275	3.2	30	1.8	2,245	3.2
**Stroke**						
Yes	2,197	3.1	76	4.6	2,121	3.1
No	66,755	93.7	1,560	93.6	65,195	93.7
Missing	2,275	3.2	30	1.8	2,245	3.2
**Activities of daily living**						
No need for personal assistance	63,052	88.5	1,300	78.0	61,752	88.8
Require some personal assistance	4,787	6.7	255	15.3	4,532	6.5
Missing	3,388	4.8	111	6.7	3,277	4.7
**Number of teeth**						
0	10,620	14.9	337	20.2	10,283	14.8
1–4	7,577	10.6	217	13.0	7,360	10.6
5–9	11,707	16.4	299	18.0	11,408	16.4
10–19	20,687	29.1	437	26.2	20,250	29.1
≥20	19,096	26.8	320	19.2	18,776	27.0
Missing	1,540	2.2	56	3.4	1,484	2.1
**Experience of pneumococcal vaccination within last five-year**						
Yes	30,174	42.4	1,016	61.0	29,158	41.9
No	39,349	55.2	565	33.9	38,784	55.8
Missing	1,704	2.4	85	5.1	1,619	2.3

Table 2 shows the proportion of participants who experienced pneumonia based on the frequency of denture cleaning and stratified by age group. Pneumonia was more prevalent among the participants who did not clean their dentures daily, especially those aged ≥75 years. Among these participants aged ≥75 years, 2.9% and 4.3% of those who did and did not clean their dentures daily, respectively, experienced pneumonia.

**Table 2:** The incidence of pneumonia within the last one year based on the frequency of denture cleaning stratified by age groups.

n (%)	All participants (n = 70,501)		65–74 years (n = 35,062)		≥75 years (n = 35,439)	
	Frequency of denture cleaning		Frequency of denture cleaning		Frequency of denture cleaning	
	Daily	Non-daily	Daily	Non-daily	Daily	Non-daily
**Incidence of pneumonia within the last one year**						
Yes	1,547 (2.3)	100 (3.0)	575 (1.7)	34 (1.9)	972 (2.9)	66 (4.3)
No	65,661 (97.7)	3,193 (97.0)	32,733 (98.3)	1,720 (98.1)	32,928 (97.1)	1,473 (95.7)

To reduce the possibility of selection bias, we estimated the propensity score for denture cleaning after MICE. After multiple imputation, the missing values of 22,020 participants were imputed. The propensity scores were predicted using the logistic regression model separately for the entire data (all participants) and stratified data (participants aged <75 years or ≥75 years) for each imputed data sets. After using stabilized ATE weight, the standardized differences of all covariates were <0.1 (Supplementary Table 1). Therefore, by using the estimated propensity score, we confirmed that the all the covariates are well balanced between those who did and did not cleaned their dentures daily. Table 3 shows the results of the logistic regression analysis using the stabilized ATE-IPW method; infrequent denture cleaning was significantly associated with the incidence of pneumonia among all participants (OR = 1.30, 95% CI = 1.01–1.68). In addition, the sensitivity analysis based on stratification by age groups showed that infrequent denture cleaning was significantly associated with the occurrence of pneumonia among those aged ≥75 years (OR = 1.58, 95% CI = 1.15–2.17). In contrast, a significant association between infrequent denture cleaning and the incidence of pneumonia was not observed among those aged <75 years (OR = 0.98, 95% CI = 0.64–1.50). However, the additive and multiplicative scale of interaction effect was not significant (Supplementary Table 2).

**Table 3:** The association between the incidence of pneumonia within the last one year and the frequency of denture cleaning.

Frequency of denture cleaning	All participants (n = 71,227)	65–74 years (n = 35,349)	≥75 y (n = 35,878)
	Stabilized ATE weighted	Stabilized ATE weighted	Stabilized ATE weighted
	OR (95% CI)	OR (95% CI)	OR (95% CI)
Daily	Ref.	Ref.	Ref.
Non-daily	1.30 (1.01–1.68)	0.98 (0.64–1.50)	1.58 (1.15–2.17)

*Note:* ATE = average treatment effect, OR = odds ratio, 95%CI = 95% confidence interval, Ref. = reference.

## Discussion

The present study revealed that infrequent denture cleaning was associated with the incidence of pneumonia within the last one year among community-dwelling older adults. This result suggests the importance of denture cleaning in reducing the risk of pneumonia among community-dwelling older adults. From the public health viewpoint, this is an important finding because the number of community-dwelling older adults is increasing in this aging world.

As mentioned in the introduction, previous studies suggested that oral hygiene including denture cleaning was associated with the incidence of pneumonia among nursing homes residents^11^; the present study showed a similar association among the community-dwelling older adults. A study conducted in nursing home reported a reduction of death due to pneumonia among older residents by oral care including denture cleaning^23^. We added that frequent denture care could reduce the incidence of pneumonia in community-dwelling older adults.

Denture plaque is composed from many species of bacteria and fungus; some of them are regarded as pathogen of pneumonia^24,25^. Infrequent denture cleaning causes accumulation of denture plaque^26^, and therefore, the possibility of the pathogens reaching the lung by aspiration might increase^27^. Consequently, it may be presumed that the pathogens from denture plaque accumulated due to infrequent cleaning were aspirated and may have increased the risk of pneumonia. In the present analysis, a strong association was observed among those aged ≥75 years, although a statistical significance was not clearly observed. With advancing age, the immune system declines^3^ and aspiration is more likely to occur in older adults rather than those who are younger^28^. The mortality rate of pneumonia is increasing among the older adults^2^. Therefore, the results of the present study are reasonable: those aged ≥75 years were more likely to develop pneumonia and the harmful effect of infrequent denture cleaning was stronger than that observed in younger participants. These results are supported by the biological explanations mentioned above. Further study considering the effect modification of dysphagia on the association between poor oral hygiene and pneumonia incidence would strengthen our explanation of the results of the present study.

The strength of this study was the inclusion of over 70,000 participants; this sample size was large enough to detect the association between infrequent denture cleaning and pneumonia. The incidence of pneumonia among community-dwelling older adults is lower than that in nursing homes where frail older adults live^29^. Therefore, it is difficult to have sufficient statistical power to detect the association in smaller epidemiological studies. This study, however, has several limitations. As this was a cross-sectional study, we could not evaluate the causal relationship between denture cleaning and pneumonia. However, it is less likely that the occurrence of pneumonia would lead to infrequent denture cleaning. In addition, the self-reported incidence of pneumonia causes reporting bias. However, the incidence of pneumonia in this study is similar to that previously reported^30^. Therefore, the reporting bias caused by the self-reporting of pneumonia was considered to be relatively small. The self-reported independent variable, denture cleaning, also created bias. A wide variety of denture cleaning methods and techniques may be used by the participants. Our questionnaire could not obtain information on the details regarding the denture cleaning methods. However, this reporting bias could widen the 95% confidence interval of our estimates. Despite this situation, there was a significant association of denture cleaning with pneumonia; therefore, we consider the present results to be robust. Furthermore, those who died because of pneumonia were not included in this study. This selection bias is considered to cause an underestimation of the association between denture cleaning and pneumonia. In the present results, the benefit of denture cleaning was remarkable among only older adults aged ≥75 years. The individuals who died from pneumonia are considered to be frail and very old^27,29^; therefore, the impact of denture cleaning on these individuals is larger than those who experienced pneumonia but are alive. The previous study revealed an association between denture wearing during sleep and pneumonia incidence among community-dwelling older adults^31^. The results of this previous study were similar to those from our study. There was a possibility of multicollinearity between denture wearing during sleep and infrequent denture cleaning. In our survey, a question about denture wearing during sleep was asked to only one-eighth of all participants (n = 8,316), so we did not include this variable in the present analysis to avoid decreasing the sample size. When analyzing this variable alone, we confirmed that the proportions of those wearing dentures during sleep were similar among those participants who did/did not clean their dentures daily (17.3% among those who cleaned their dentures daily and 18.5% among those who did not clean their dentures daily wore dentures during sleep; chi-square test, *p* = 0.544). Therefore, infrequent denture cleaning is associated with pneumonia incidence and is independent of denture wearing during sleep.

## Conclusion

The present study revealed that infrequent denture cleaning was associated with the incidence of pneumonia within the last one year among community-dwelling older adults. Daily cleaning of dentures may reduce the risk of pneumonia among community-dwelling older adults. In the chair side, dental professionals need to instruct their patients to keep their dentures clean to prevent pneumonia. Even for community-dwelling older adults, dental professionals should pay more attention to oral hygiene for pneumonia prevention.

## Supplementary information

Supplementary Material

## Data Availability

All data needed to evaluate the conclusions in the paper are present in the paper and/or the Supplementary Materials. The JAGES data used in this study will be made available upon request. The authors require the applicant to submit an analysis proposal to be reviewed by an internal JAGES committee to avoid duplication. Confidentiality concerns prevent us from depositing our data in a public repository. Proposals submitted by outside investigators will be discussed during the monthly investigators’ meeting to ensure that there is no overlap with ongoing analyses. If approval to access the data is granted, the JAGES researchers will request the outside investigator to help financially support our data manager’s time to prepare the data for outside use.
