# Supplementary material for: Infrequent Denture Cleaning Increased the Risk of Pneumonia among Community-dwelling Older Adults: A Population-based Cross-sectional Study
Source: Sci Rep. 2019 Sep 24;9:13734. doi: 10.1038/s41598-019-50129-9 (PMC6760190; doi:10.1038/s41598-019-50129-9)
Supplement: Supplementary file 1 — Supplementary Material [file 41598_2019_50129_MOESM1_ESM.pdf]

Supplementary Material for 'Infrequent Denture Cleaning Increased the Risk of Pneumonia among Community-dwelling Older Adults: A Population-based Cross-sectional Study.'

Taro Kusama,[1] Jun Aida,[1*] Tatsuo Yamamoto,[2] Katsunori Kondo,[3,4] Ken Osaka[1]

1) Department of International and Community Oral Health, Tohoku University Graduate School of Dentistry, Aoba-ku, Sendai, Miyagi, Japan

2) Department of Disaster Medicine and Dental Sociology, Graduate School of Dentistry, Kanagawa Dental University, Yokosuka, Kanagawa, Japan

3) Department of Social Preventive Medical Sciences, Center for Preventive Medical Sciences, Chiba University, Chuo Ward, Chiba-shi, Chiba, Japan

4) Department of Gerontological Evaluation, Center for Gerontology and Social Science, National Center for Geriatics and Gerontology, Obu city, Aichi, Japan

*Corresponding author

Jun Aida

Department of International and Community Oral Health, Tohoku University Graduate School of Dentistry, Sendai, Japan.

Address: 4-1, Seiryo-machi, Aoba-ku, Sendai, Miyagi, 980-8575, Japan.

E-mail: j-aida@umin.ac.jp

Tel: +81-22-717-7639

Supplementary Table 1. Standardized differences of covariate before and after stabilized average treatment effect weighting

	All participants (n = 71,227)		65 - 74y (n = 35,349)		≥75y (n = 35,878)	
	Raw	Weighted	Raw	Weighted	Raw	Weighted
Age						
65 - 69y	0.115	0.021	0.124	0.045	—	—
70 - 74y	0.031	0.021	0.124	0.045	—	—
75 - 79y	0.132	0.023	—	—	0.157	0.026
80 - 84y	0.008	0.019	—	—	0.041	0.024
≥85y	0.069	0.009	—	—	0.144	0.004
Sex	0.473	0.051	0.546	0.029	0.393	0.087
Education						
≤9y	0.100	0.019	0.048	0.003	0.181	0.017
10 - 12y	0.139	0.020	0.104	0.008	0.196	0.023
≥13y	0.043	0.002	0.066	0.011	0.002	0.005
Equivalent income						
<1,000,000JPY	0.090	0.014	0.080	0.012	0.111	0.016
1,000,0000 - 1,999,999 JPY	0.020	0.005	0.047	0.010	0.014	0.014
2,000,0000 - 2,999,999 JPY	0.042	0.011	0.041	0.017	0.043	0.004
3,000,0000 - 3,999,999 JPY	0.042	0.012	0.045	0.017	0.043	0.008
≥4,000,000 JPY	0.036	0.020	0.055	0.015	0.015	0.016
Smoking status						
never	0.335	0.047	0.397	0.039	0.257	0.067
quite	0.158	0.040	0.165	0.027	0.147	0.067
current	0.244	0.012	0.273	0.014	0.189	0.005
Dementia	0.184	0.005	0.122	0.005	0.242	0.007
Stroke	0.098	0.013	0.090	0.000	0.111	0.023
Activities of daily living	0.272	0.023	0.198	0.007	0.366	0.029
Number of teeth						
0	0.163	0.007	0.182	0.015	0.172	0.002
1 - 4	0.000	0.002	0.024	0.037	0.015	0.022
5 - 9	0.029	0.020	0.022	0.006	0.032	0.049
10 - 19	0.114	0.022	0.133	0.029	0.102	0.011
≥20	0.003	0.034	0.002	0.036	0.029	0.041
Experience of pneumococcal vaccination within last five-year	0.217	0.012	0.128	0.001	0.292	0.016

Supplementary Table 2. Effect modification of frequency of denture cleaning on the incidence of pneumonia within last one-year by age (n = 71,227)

	Cleaning denture daily		Cleaning denture non-daily		aORs (95%CIs) for frequency of denture cleaning within strata of age group
	n with/without the incidence of pneumonia	aOR (95% CI)	n with/without the incidence of pneumonia	aOR (95% CI)	
65-74y	575/32,723	1.00	34/1,720	0.99 (0.69 - 1.40)	1.03 (0.72 - 1.47)
≥75y	972/32,928	1.20 (1.08 - 1.35)	66/1,473	1.65 (1.26 - 2.16)	1.37 (1.06 - 1.78)

Measure of effect modification on additive scale: RERI (95%CI) = 0.46 (-0.08 - 1.00); *P* = 0.098

Measure of effect modification on multiplicative scale: aOR (95%CI) = 1.38 (0.90 - 2.15); *P* = 0.141

aORs are adjusted for age, sex, education, equivalent income, smoking status, dementia, stroke, ADL, number of teeth, and experience of pneumococcal vaccination within last five-year

Note: aOR = adjusted odds ratio, 95%CI = 95% confidence interval, RERI = relative excess risk due to interaction, ADL = Activities of daily living
